# Efficacy and mechanisms of vagus nerve stimulation in irritable bowel syndrome: a comprehensive literature review

**DOI:** 10.3389/fimmu.2026.1769070

**Published:** 2026-03-11

**Authors:** Yiwen Quan, Tao Zhou

**Affiliations:** 1 Department of Gastroenterology, Qilu Hospital of Shandong University, Jinan, Shandong, China; 2 Department of Geriatric Medicine & Laboratory of Gerontology and Anti-Aging Research, Qilu Hospital of Shandong University, Jinan, Shandong, China

**Keywords:** gut-brain axis, intestinal microbiota, irritable bowel syndrome, vagus nerve stimulation, visceral hypersensitivity

## Abstract

Irritable bowel syndrome (IBS) is a common non-organic functional gastrointestinal disorder. Recently, vagus nerve stimulation (VNS) has been a novel therapeutic strategy for the treatment of IBS. This review provided an overview of the mechanisms of VNS in IBS and clinical applications. Studies about VNS in the treatment of IBS were systematically retrieved from PubMed, EMbase and CNKI databases. Mechanically, pre-clinical evidence highlights VNS as a multifaceted therapy for IBS, including reducing inflammation via α7 nicotinic acetylcholine receptor-mediated suppression of TNF-α, alleviating visceral hypersensitivity, increasing colorectal distension thresholds, enhancing motility through M3 receptor activation and gastric emptying, restoring gut microbiota diversity via elevating bifidobacterium abundance and short-chain fatty acids, and improving intestinal barrier integrity. Consistent with its mechanism, the reduced inflammation biomarkers, improved metabolism content, increased genus bifidobacterium, and improved intestinal barrier integrity are confirmed in clinical patients with IBS after VNS therapy. Besides, clinical studies reveal that VNS can increase the complete spontaneous bowel movements per week, decrease the visual analog scale for abdominal pain and IBS symptom score, improve rectoanal inhibitory reflex, rectal sensation, and improve sustained quality of life. In summary, noninvasive VNS is an effective and novel therapy option for patients with IBS, and its integrative effects are multi-factorial.

## 1 Introduction

Irritable bowel syndrome (IBS) is a common non-organic functional gastrointestinal disease affecting approximately 5% to 10% of the population, characterized by recurrent abdominal pain, bloating, and irregular bowel habits. According to different clinical manifestations, it can be divided into constipation type (IBS-C), diarrhea type (IBS-D), mixed type (IBS-M), and indefinite type (IBS-I) (1). The potential mechanism of IBS is multifactorial, and most existing medical therapies can only relieve symptoms rather than provide a cure. Moreover, these drugs and diet therapy supported by low-quality evidence, including low-FODMAP (fermentable oligosaccharides, disaccharides, monosaccharides, and polyols) diet and enteric-directed hypnotherapy (2, 3), often exhibit limited efficacy and many adverse effects.

In recent years, studies demonstrate that vagus nerves play key roles in regulating gastrointestinal sensitivity, movement, and immune function, and their dysfunction is associated with a variety of gastrointestinal diseases (4). Besides, a recent study verifies that vagus nerve stimulation (VNS), as a bioelectric medical treatment, could improve gastrointestinal motility, reduce inflammatory response, and regulate intestinal microbiota. VNS is increasingly explored in the clinical treatment of IBS, expected to become a promising non-drug therapy for gastrointestinal diseases (5). This review aims to provide an overview of the mechanisms of VNS in IBS and clinical applications.

## 2 Overview of the vagus nerve and its role in gastrointestinal disease

The vagus nerve is a mixed nerve with 20% efferent and 80% afferent fibers: Afferent fibers carry information from various parts of the body to the brain; efferent fibers control autonomic functions such as heart rate, blood pressure, and movement and secretion of the gastrointestinal tract (6–8). In rats, the vagus nerve innervates the entire gastrointestinal tract except the rectum, while its dominant area of the gastrointestinal tract is controversial in humans. Some studies show the vagus nerve innervates the digestive tract until the splenic flexure of the colon, while others demonstrate the vagus nerve innervates the digestive tract in humans (6–8).

The afferent fibers of the vagus nerve are distributed in various layers of the digestive wall but do not directly contact the intestinal microflora. Instead, they perceive microbial signals indirectly through the diffusion of bacterial compounds and other cell mediators. Enteroendocrine cells (EECs) (such as enterochromaffin cells) can detect microbial signals and interact with afferent fibers of the vagus nerve to regulate gastrointestinal function by releasing hormones and neurotransmitters like serotonin (5-HT) and cholecystokinin (CCK) (9, 10). EECs can sense the changes in nutrients, osmotic pressure, and pH in the intestinal space. As mucosal taste cells, EECs not only act on adjacent intestinal epithelial cells through paracrine but also activate receptors in the enteric nervous system, vagus nerve, and spinal cord afferent pathways, playing a key role in the information transmission between the intestinal and nervous systems (9). Studies have shown that patients with IBS have abnormal serotonin levels, and this abnormality is associated with abnormal expression of serotonin transporter (SERT) (11). The release of 5-HT remains unchanged in IBS patients, but the decreased expression of SERT may impair the uptake of 5-HT, which increases the availability of 5-HT in the mucosa, enhancing reflex activity and triggering IBS-D. Conversely, long-term exposure to high levels of 5-HT may also desensitize 5-HT’s receptors, triggering IBS-C (12).

Lack of vagal tone has been identified as a pathophysiological feature of IBS, which is due to an imbalance in the regulation of the autonomic nervous system (13). Low vagal tone is often associated with abnormal bowel movements in IBS patients, such as reduced bowel motility in IBS-C patients. Studies have shown that the combination of electrical stimulation and physiological regulation of vagal tone can enhance gastroduodenal motility and reduce the sensitivity of physical pain (14). Some studies used ultrasound to evaluate the dimensional changes of the vagus nerve, which found that the electrophysiological activity of VN in patients with IBS changed in the resting state, but there were no significant changes in diameter or area. This indicates that the changes of the vagus nerve in IBS are mainly reflected in function rather than structure (15).

## 3 The mechanisms of VNS in IBS

**Figure 1:**
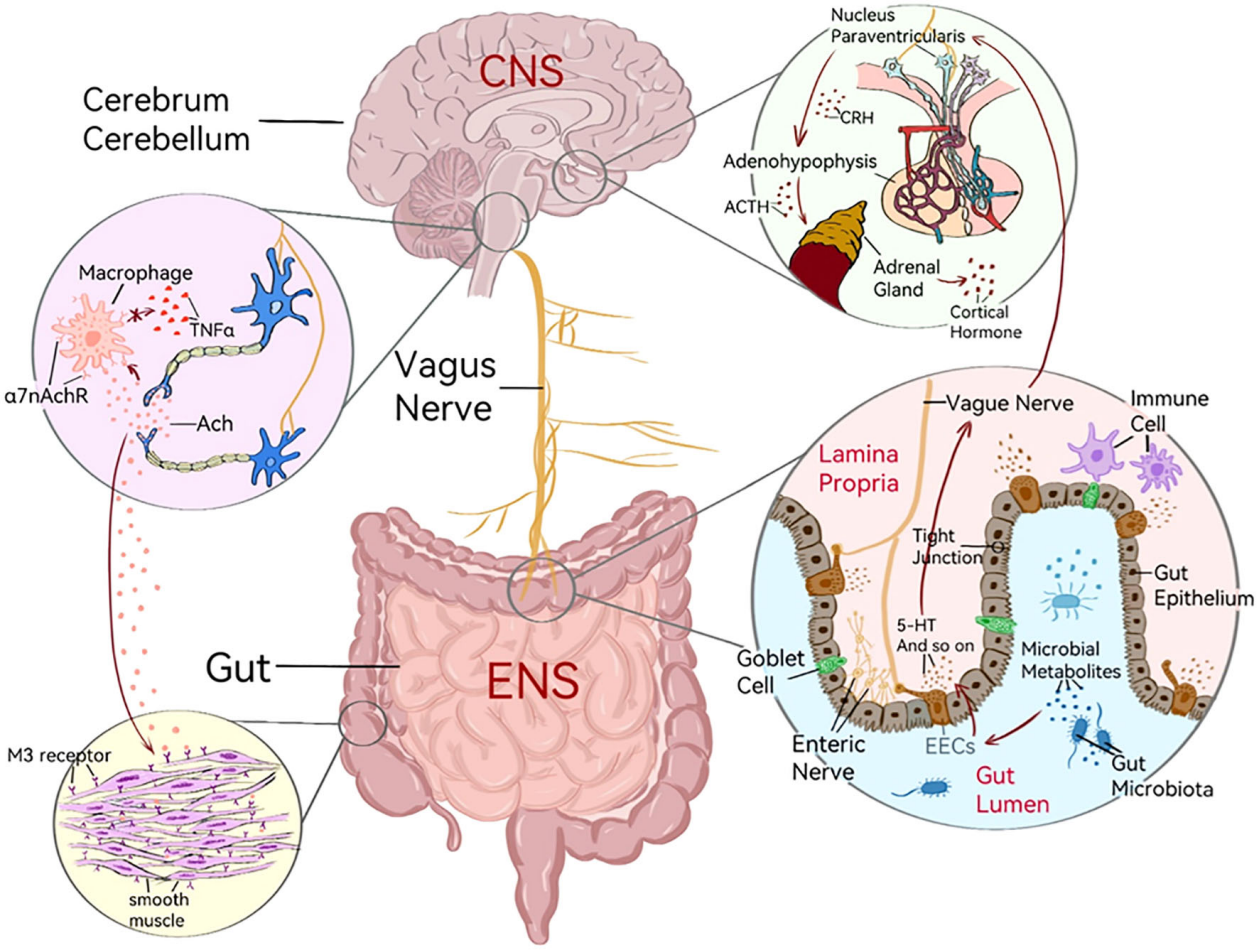
The mechanisms of VNS in IBS. Vagus nerve connects the enteric nervous system (ENS) of the gastrointestinal tract to the central nervous system (CNS) of the brain; HPA axis: the nucleus paraventricularis in hypothalamus secrete corticotropin releasing hormone (CRH), stimulating adenohypophysis to secrete adrenocorticotropic hormone (ACTH) and finally making the adrenal gland secrete cortical hormone; When the gut microbiota is disturbed, the gut microbes will release microbial metabolites, send out microbial signals, and then stimulate the proliferation of enteroendocrine cells (EECs) and release 5-HT to stimulate the afferent fibers of VN; Pro-inflammatory cytokines released by immune cells after stimulation can activate afferent fibers of VN, thereby activating the HPA axis; Gut epithelium form the intestinal barrier through tight junctions and are involved in maintaining the integrity of the intestine; Acetylcholine (Ach) combines with the α7 nicotinic Ach receptor (α7nAChR) on macrophages, thereby inhibiting the production of proinflammatory cytokines by macrophages like Tumor Necrosis Factor (TNF-α); Acetylcholine released from endings of VN acts on M3 receptors on smooth muscle membrane, which can lead to contraction of gastrointestinal smooth muscle.

Previous studies verify that VNS plays an essential role in IBS, and the potential mechanisms are summarized in **Figure 1**.

### 3.1 Gut-brain axis

GBA is a bidirectional communication system that connects the enteric nervous system of the gastrointestinal tract to the central nervous system of the brain (16). The enteric nervous system can independently regulate gastrointestinal peristalsis, nutrient absorption, and other key processes, and the vagus nerve is a key component of GBA. The vagus nerve can transmit intestinal information to the brain and regulate intestinal function; gut microbiota, which is involved in bidirectional signaling between the gut and the brain. The vagus nerve also influences neural activity and immune responses by producing bioactive compounds of metabolism and vitamin synthesis (5).

Patients with IBS often present with functional imbalances in the GBA, resulting in abnormal gastrointestinal motility, visceral hypersensitivity, intestinal inflammation, and intestinal microecological imbalances (5, 11). Studies have shown that VNS can improve the conduction of the vagus nerve and restore the balance of the GBA by enhancing the conduction function of the vagus nerve, thereby improving the movement and secretion function of the gastrointestinal tract, reducing the chronic low-grade inflammation of the intestinal tract, and thus alleviating the symptoms of IBS (17).

### 3.2 Inflammation inhibition

The afferent fibers of the vagus nerve play an anti-inflammatory role by activating the hypothalamic-pituitary-adrenal (HPA) axis and prompting the adrenal glands to release corticosteroids with anti-inflammatory properties (7). Studies have shown that peripheral injection of lipopolysaccharide LPS can activate afferent fibers of the vagus nerve, thus activating A2 noradrenergic neurons in the nucleus tractus solitarii, promoting the release of corticotropin-releasing factor, stimulating the adenohypophysis to release adrenocorticotropic hormone, and finally making the adrenal gland secrete corticosteroid hormone to inhibit peripheral inflammation (6). In addition, pro-inflammatory cytokines released by immune cells after stimulation can communicate with the brain through neural and humoral pathways to activate afferent fibers of the vagus nerve, thereby activating the HPA axis (7). The afferent fibers of the vagus nerve activate the central autonomic network and regulate sympathetic nerve activity through a descending pathway, thereby affecting the inflammatory response. For example, brain regions such as the hypothalamic paraventricular nucleus are involved in the regulation of inflammation by regulating the sympathetic nerve (6).

The efferent fibers of the vagus nerve play an anti-inflammatory role through the cholinergic anti-inflammatory pathway (7). Inflammatory mediators activate the afferent fibers of the vagus nerve, transmit inflammatory information to the central nervous system, and then activate efferent fibers. Acetylcholine released from the cholinergic anti-inflammatory pathway interacts with α7 nicotinic acetylcholine receptor (α7nAChR) on macrophages and leads to inhibition of proinflammatory cytokine production by macrophages, such as tumor necrosis factor (TNF-α) (18). In a rat model of IBS established by acetic acid irrigation combined with chronic acute stress, VNS improved depressive behavior, abnormal fecal output, and excessive abdominal withdrawal reflex in IBS rats, the mechanism of which may be related to α7nAChR reducing levels of inflammatory cytokines (19). The spleen plays an important role in the cholinergic anti-inflammatory pathway. The vagus nerve is connected to the splenic sympathetic nerve and regulates the secretion of TNF-α by splenic macrophages through α7nAChR. In addition, the vagus nerve also has a regulatory effect on splenic lymphocytes, affecting the proliferation of T cells, migration of B cells, and production of antibodies (7).

### 3.3 Pain relief

Studies have shown that percutaneous electrical nerve field stimulation (PENFS) significantly reduces abdominal pain scores in adolescents with IBS, improving diarrhea and constipation​ (20). In addition, some animal experiments showed that visceral pain thresholds were decreased and responses increased in rats after chronic subphrenic vagotomy. Besides, electrical stimulation of afferent fibers of the vagus nerve was also confirmed to inhibit visceral motor response, increase visceral pain threshold, and alleviate pain response (21). Chronic pain may lead to impaired decision-making ability, while chronic VNS can increase the proportion of rats with good decision-making, improving cognitive ability in patients with chronic visceral pain by regulating neuronal synchronization (22). It was suggested that transcutaneous auricular VNS (taVNS) with a stimulation frequency between 20Hz and 30Hz may aid in pain relief (23).

### 3.4 Improvement in visceral hypersensitivity

Visceral hypersensitivity is also a key feature of IBS, characterized by enhanced perception of mechanical stimulation of the intestine, reduced pain threshold of colon dilation, and susceptibility to abdominal pain (11). Studies have shown that elevated levels of substances such as prostaglandin E2 in colon biopsies of IBS patients may activate pain signaling and trigger visceral hypersensitivity (11). VNS can reduce excessive pain perception in the internal organs. Non-invasive VNS, such as taVNS, has been shown to alleviate visceral hypersensitivity in both animal and clinical studies. An animal study showed that taVNS increased colorectal dilation thresholds and reduced abdominal withdrawal reflex scores in mice, suggesting that taVNS can improve visceral hypersensitivity (24).

### 3.5 Adjustment to gastrointestinal motility

Studies have shown that taVNS can increase the frequency of bowel movements, reduce abdominal pain and constipation, and improve quality of life in patients with IBS-C (25). VNS enhances the outflow activity of the vagus nerve, cooperates with the sacral nerve through the central-vagus pathway, and improves the coordination of rectal-anal function (25). In a mouse with an IBS model, VNS increased fecal particle count, wet weight, and water content, accelerated gastric emptying rate and intestinal peristalsis frequency, and improved constipation (24). Acetylcholine from the vagus nerve acts on M3 receptors on the smooth muscle membrane, which can lead to the contraction of gastrointestinal smooth muscle. Electroacupuncture stimulation, such as VNS, also enhances the positive expression of M3 receptors in intestinal smooth muscle, while reducing the expression of 5-HT_2A_ (One subtype of 5-HT) receptors in the colon. This can provide inhibitory effects for hyperactive intestinal motility (such as IBS-D), and excitatory effects for hypotonic intestinal motility (such as IBS-C), demonstrating the characteristic of bidirectional positive regulation (26).

### 3.6 Adjustment to intestinal flora

By changing the intestinal microenvironment, VNS can also regulate the intestinal flora and enhance the number of beneficial bacteria such as bifidobacterium and lactic acid bacteria (24). Clinical studies have found that taVNS treatment increased the intestinal flora of bifidobacteria and the concentration of short-chain fatty acids such as butyric acid, propionic acid, and acetic acid significantly, which could alleviate the inflammatory response associated with IBS (27). These results suggest that regulation of the gut microbiota plays an important role in taVNS alleviating symptoms of IBS.

### 3.7 Maintenance of the intestinal barrier’s integrity and reduction of permeability

Impairment of the intestinal barrier often leads to ectopic microbial composition, disturbances, and low-grade inflammation throughout the body, leading to IBS (18). Among them, enteric glial cells (EGCs) are the main cells of the enteric nervous system, which is associated with the inflammatory response. EGCs can secrete S-nitroso-glutathione and other molecules, increase the expression of tight junction proteins (Occludins、Members of the claudin family、Zonula-occludin proteins (ZO proteins), specifically ZO-1、Phosphorylated myosin light chain、Myosin light chain kinase), and protect the epithelial barrier. In addition, EGCs can regulate the infiltration of immune cells and play an important role in maintaining the integrity of the intestinal barrier and the balance of immune response (18). Although the vagus nerve cannot directly innervate intestinal epithelium, it can affect EGCs to regulate intestinal permeability via communication with the enteric nervous system through nicotinic cholinergic signals (18). In a mouse model of traumatic brain injury, VNS can prevent the increase of intestinal permeability and intestinal damage with reduced intestinal level of TNF-α (28). While VNS pretreatment before or within 90 minutes after injury can effectively protect the intestinal epithelial barrier, indicated that there is a “treatment window” for VNS in improving symptoms of IBS (28). It was also found that the vagus nerve participates in the activation of central adenosine A2B receptors and thus improves hyperpermeability of the colon, and the activation of the vagus nerve can improve intestinal leakage and reduce intestinal permeability (29). At present, the vagus nerve-mediated mechanism of IBS-C has been clearly identified, but the complete potential molecular mechanism underlying IBS-D remains unclear. Most of the reports focus on improving abdominal pain and quality of life.

## 4 Clinical application of VNS in IBS

VNS can be divided into invasive vagus nerve stimulation (iVNS) and transcutaneous vagus nerve stimulation (tVNS). iVNS requires implantation of stimulating electrodes by surgery, while tVNS only requires electrical stimulation through the skin (30). Among them, tVNS can be divided into cervical vagus nerve stimulation in the neck (tcVNS), auricular vagus nerve stimulation in the ear (taVNS), and percutaneous electrical nerve field stimulation (PENFS), which have the potential function of regulating pain and inflammation and are expected to become a non-drug therapy for the treatment of gastrointestinal diseases (4). To systematically retrieve the studies, we searched and eventually included six clinical studies of VNS in IBS on PubMed, Embase, and CNKI from the inception to May 2025. The search strategy is shown in **Supplementary File 1**. These studies evaluated the effect of VNS in IBS involving multiple clinical outcomes. The findings of these clinical studies are summarized in **Table 1**; the detailed outcome data are described in **Table 2**, and the stimulation method, site, frequency, intensity, pulse width, cycle, and duration are presented in **Table 3**.

**Table 1:** Study features and main findings.

Study ID	Study design	Stimulus method	No. of patients		Main findings	
			VNS	Sham		
Shi et al.	RCT	taVNS	21	19		Compare to Sham-VNS: a. VNS decreased the VAS pain score by 64% (3.1 ± 2.2 versus 1.1 ± 1.1, P = 0.001) b. VNS made the number of CSBMs/weeks triple (0.9 ± 0.9 versus 2.8 ± 2.2, P = 0.001) c. VNS decreased the IBS-SSS significantly (289.5 ± 94.4 versus 197.1 ± 39.6, P = 0.001) d. VNS increased BSFS score significantly (1.8 ± 1.1 versus 3.7 ± 1.3, P<0.001) e. VNS increased IBS-QOL score significantly (69.5 ± 21.2 versus 83.2 ± 12.5, P = 0.020)
Liu et al.	RCT	taVNS	20	20		Compare to Sham-VNS: a. VNS decreased the VAS pain score (5.25 ± 1.25 versus 2.90 ± 0.79, P<0.001) b. VNS increased the number of CSBMs/weeks significantly (4.00 ± 1.30 versus 2.80 ± 1.15, P = 0.004) c. VNS decreased the IBS-SSS significantly (200.00 ± 53.31 versus 294.50 ± 35.76, P<0.001) d. VNS increased BSFS score significantly (3.00 ± 0.79 versus 1.80 ± 0.62, P<0.001) e. VNS increased IBS-QOL score significantly (72.60 ± 23.28 versus 96.85 ± 22.42, P = 0.002)
Bora et al.	RCT	PENFS	20	NA		Compared to baseline: a. VNS decreased the IBS-SSS significantly (256.0 ± 19.6 versus 150.0 ± 19.6, P<0.0001) b. VNS decreased VSI score significantly (45.0 ± 3.8 versus 34.0 ± 3.8, P = 0.0003) c. VNS decreased FDI score (geometric mean: 9 [95% CI 6-14] versus geometric mean: 4 [95% CI 3–6]) d. α diversity was higher in the “mild responders” based on a significantly higher inverse Simpson index compared to non-responders at week 4(P = 0.033), while β diversity between pre-therapy and post-therapy in “mild”, “robust”, and “excellent responders” compared to non-responders was not different
Krasaelap et al.	RCT	PENFS	27	23		a. 59% of PENFS-treated patients experienced a 30% or more reduction in their most severe abdominal pain, compared to only 26% of patients who received fake stimulation (P = 0.024). b. The patients who received PENFS had a composite pain median score of 7.5 (interquartile range [IQR], 3.6-14.4) versus 14.4 for the sham group (IQR, 4.5-39.2) (P = 0.026) and a usual pain median score of 3.0 (IQR, 3.0-5.0) versus 5.0 in the sham group (IQR, 3.0-7.0) (P = 0.029) c. A symptom response scale score of 2 or more was observed in 82% of patients who received PENFS versus 26% of patients in the sham group (P ≤ 0.001)
Chogle et al.	RCT	PENFS	292	NA		a. Baseline (n=288) median (IQR) child‐reported API scores decreased from 2.68 (1.84, 3.58) to 1.99 (1.13, 3.27) at 3 weeks (P<0.001) and 1.81 (0.85, 3.20) at 3 months (n=75, P<0.001) b. Child median (IQR) NSS scores similarly improved from baseline, persisting at three (n=74, P<0.001) and 6 months later (n=55, P<0.001) c. Child median (IQR) FDI scores decreased across time from baseline (n=290) 20 (9.0, 29.0) to 12.0 (4.0, 24.0) at 3 weeks (n=209, P<0.001)
Chen et al.	RCT	taVNS	10	10		Compare to Sham-VNS: a. VNS decreased the IBS-SSS significantly (219.70 ± 72.30 versus 90.00 ± 39.83, P<0.05) b. VNS decreased the HAMD significantly (12.20 ± 5.67 versus 7.80 ± 6.80, P<0.05) c. VNS decreased the HAMA significantly (10.10 ± 4.31 versus 4.90 ± 3.14, P<0.05)
Mion et al.	RCT	taVNS	9	NA		Compared to baseline: a. VNS decreased the IBS-SSS significantly (336.0 ± 26.0 versus 231.0 ± 26.0, P = 0.0084, at 3 months) (336.0 ± 26.0 versus 246.0 ± 26.0, P = 0.0209, at 6months) b. VNS decreased the CES-D significantly (21.0 ± 4.0 versus 19.0 ± 5.0, at 3 months) (21.0 ± 4.0 versus 19.0 ± 4.0, at 6 months) c. VNS decreased the PSS significantly (25.0 ± 7.0 versus 21.0 ± 9.0 at 3 months) (25.0 ± 7.0 versus 19.0 ± 6.0 at 6 months) d. VNS decreased the STAI significantly (44.0 ± 12.0 versus 42.0 ± 10.0 at 3 months) (44.0 ± 12.0 versus 37.0 ± 11.0 at 6 months)

VAS, visual analog scale; CSBMs/weeks, complete spontaneous bowel movements per week; IBS-SSS, IBS symptom severity scale, used to measure the severity of IBS; BSFS, Bristol Stool Form Scale; IBS-QOL, IBS quality-of-life questionnaire; VSI, visceral sensitivity index; API, abdominal pain index; NSS, nausea severity scale; FDI, functional disability inventory; HAMD, Hamilton depression Scale; HAMA, Hamilton Anxiety Scale; CES-D, Center for Epidemiologic Studies-depression scale; PSS, Perceived stress scale; STAI, State-trait anxiety inventory.

**Table 2:** Outcome data.

Outcome Indicator	Study ID	VNS		Sham-VNS	
		No. of patients	Data	No. of patients	Data
VAS pain	Shi et al.	21	1.1 ± 1.1	19	3.1 ± 2.2
	Liu et al.	20	2.90 ± 0.79	20	5.25 ± 1.25
	Bora et al.	20	150 ± 19.6	NA	256.0 ± 19.6
	Krasaelap et al.	27	3[IQR,3.0-5.0]	23	5[IQR,3.0-7.0]
	Chogle et al.	292	NA	NA	NA
	Chen et al.	10	NA	10	NA
	Mion et al.	9	NA	NA	NA
IBS-SSS	Shi et al.	21	197.1 ± 39.6	19	289.5 ± 94.4
	Liu et al.	20	200.00 ± 53.31	20	294.50 ± 35.76
	Bora et al.	20	NA	NA	NA
	Krasaelap et al.	27	NA	23	NA
	Chogle et al.	292	NA	NA	NA
	Chen et al.	10	90.00 ± 39.83	10	219.70 ± 72.30
	Mion et al.	9	231.0 ± 26.0 (3 months) 246.0 ± 26.0 (6 months)	NA	336.0 ± 26.0
CSBMs/weeks	Shi et al.	21	2.8 ± 2.2	19	0.9 ± 0.9
	Liu et al.	20	4.00 ± 1.30	20	2.80 ± 1.15
	Bora et al.	20	NA	NA	NA
	Krasaelap et al.	27	NA	23	NA
	Chogle et al.	292	NA	NA	NA
	Chen et al.	10	NA	10	NA
	Mion et al.	9	NA	NA	NA
IBS-QOL	Shi et al.	21	83.2 ± 12.5	19	69.5 ± 21.2
	Liu et al.	20	96.85 ± 22.42	20	72.60 ± 23.28
	Bora et al.	20	NA	NA	NA
	Krasaelap et al.	27	NA	23	NA
	Chogle et al.	292	NA	NA	NA
	Chen et al.	10	NA	10	NA
	Mion et al.	9	NA	NA	NA
BSFS	Shi et al.	21	3.7 ± 1.3	19	1.8 ± 1.1
	Liu et al.	20	3.00 ± 0.79	20	1.80 ± 0.62
	Bora et al.	20	NA	NA	NA
	Krasaelap et al.	27	NA	23	NA
	Chogle et al.	292	NA	NA	NA
	Chen et al.	10	NA	10	NA
	Mion et al.	9	NA	NA	NA
Anxiety and Depression	Shi et al.	21	38.7 ± 5.6	19	47.9 ± 9.0
	Liu et al.	20	42.6 ± 8.1	20	50.7 ± 11.1
	Bora et al.	20	NA	NA	NA
	Krasaelap et al.	27	NA	23	NA
	Chogle et al.	292	NA	NA	NA
	Chen et al.	10	HAMA: 4.90 ± 3.14 HAMD: 7.80 ± 6.80	10	HAMA: 10.10 ± 4.31 HAMD: 12.20 ± 5.67
	Mion et al.	9	STAI: 44.0 ± 12.0 (3 months) 37.0 ± 11.0 (6 months) CES-D: 19.0 ± 5.0 (3 months) 19.0 ± 4.0 (6 months)	NA	STAI: 44.0 ± 12.0 CES-D: 21.0 ± 4.0

**Table 3:** The key treatment parameters.

Study ID	Stimulation method	Specific stimulation method	Stimulation site	Stimulation frequency	Stimulation intensity	Stimulation pulse width	Stimulation cycle	Stimulation duration
Shi et al.	taVNS	Watch-size digital stimulator (SNM-FDC01), one pair of electrodes	taVNS group: bilateral auricular cymba concha; Sham group: elbow area	25HZ	0–2 mA (maximum level tolerated by the subject)	0.5 ms	2 seconds on, 3 seconds off	Twice a day (8 a.m. and 8 p.m.), 30 minutes each time, for 4 consecutive weeks
Liu et al.	taVNS	Watch-size digital stimulator (SNM-FDC01), unilaterally positioned electrodes	taVNS group: auricular cymba concha and cavity of concha; Sham group: earlobe and antihelix	25HZ	0–2 mA (maximum level tolerated by the subject)	0.5 ms	2 seconds on, 3 seconds off	Once a day (3 p.m.), 30 minutes each time, for 4 consecutive weeks
Bora et al.	PENFS	Battery-powered, low-voltage (3.2 V) neurostimulation device (Neuraxis)	External ear	Not specified	3.2 V	Not specified	Not specified	5 days per week, for 4 consecutive weeks (daily wearing duration not specified)
Krasaelap et al.	PENFS	Neurostimulation device (Neuro-Stim), four electrode wires (three with sterile 2-mm titanium needles and one ground lead placed on the earlobe)	Adjacent to vascular branches of the external ears	Alternating frequencies (1 Hz and 10 Hz)	3.2 V	1 ms	2 hours on, 2 hours off	5 days per week, for 4 consecutive weeks (total wearing duration: 120 hours/5 days)
Chogle et al.	PENFS	Four percutaneously placed electrodes (three frontal and one dorsal)	Adjacent to auricular neurovascular bundles	Alternating frequencies (1–10 Hz)	3.2 V	Not specified	Not specified	5 days per week, for 4 consecutive weeks (worn day and night, device replaced weekly)
Chen et al.	taVNS	Huotaowei Model SDZ-II Electronic Acupuncture Therapy Instrument; Modified 2-3mm Circular Tin Sheet Electrode	taVNS group: Left auricular cavity; Sham group: Oral administration of the intestinal distress formula granules for treatment	20Hz	3–6 mA (maximum level tolerated by the subject)	Not specified	Not specified	Once a day, 30 minutes each time, for 4 consecutive weeks
Mion et al.	taVNS	Urostim 2 stimulator and ear plug	The concha of the left ear	30HZ	0.5–20 mA	250 μs	Applied continuously	5 days per week (preferably before bed time), 3 hours each time, for 6 consecutive weeks

Anatomically speaking, there are differences in the vagus nerve colon innervation range between animal models (such as rats) and humans (the animal model innervates a greater area, while the human situation is controversial), but this difference does not affect the clinical efficacy of VNS. The main reasons for this are twofold: First, although the rectum and parts of the distal colon in humans have no direct vagus nerve innervation, VNS can activate the vagus afferent fibers, project to the central solitary nucleus (NTS), thereby enhancing the output activity of the sacral nerves and regulating the functions of the distal colon and rectum, achieving the therapeutic effect (25, 31). Second, human VNS relies more on the multi-pathway synergy after central integration (such as the cholinergic anti-inflammatory pathway), and does not only depend on the direct vagus nerve control, and the core mechanisms such as anti-inflammatory (inhibiting TNF-α, IL-6), regulation of neurotransmitters (reducing 5-HT), and improvement of intestinal motility are consistent across species (3, 4, 19). These alternative mechanisms make up for the lack of direct vagus nerve innervation in the distal intestine and can effectively improve IBS symptoms (constipation, pain).

In a randomized controlled trial by Shi et al., 40 patients aged 18–75 years who met the Rome IV diagnostic criteria for IBS-C were enrolled. They were randomly assigned to either the taVNS group or the sham-taVNS group (pseudo-stimulation). The taVNS treatment was performed at the auricular cymba concha, and the sham point was at the elbow area. Patients in both groups were treated twice a day for 30 minutes each time, lasting for 4 weeks. Compared to sham-taVNS, taVNS increased the number of complete spontaneous bowel movements per week (CSBMs/week) (*P* = 0.001), decreased the visual analog scale (VAS) pain score (*P* = 0.001), improved quality of life (*P* = 0.020), and decreased IBS symptom score (*P* = 0.001). The vagal activity was weakly correlated with the number of CSBMs/weeks (*r* = 0.391; *P* = 0.010) and the VAS pain score (*r* = –0.347; *P* = 0.025) (31). Consistently, another randomized controlled trial including 40 adult patients with IBS-C revealed that taVNS was associated with significantly improved VAS pain score (P < 0.001), IBS symptom severity scale (IBS-SSS) (P < 0.001), the number of CSBMs/weeks (P< 0.001), and IBS Quality of Life score (IBS-QOL) (P < 0.001) (27). Similarly, a pilot study involving 9 women with moderate to severe IBS found that after 6 months of continuous high-frequency electrical stimulation using taVNS, the IBS-SSS scores of the patients were significantly reduced. Most of the patients reported improvement in their symptoms and a decrease in the use of antispasmodic and laxative medications (32).

A prospective study with 20 female adolescents with IBS-C showed that PENFS therapy, another approach of VNS, could decrease the IBS symptom severity scale, visceral sensitivity index, and functional disability inventory scores significantly. Notably, no intra- or interindividual microbiome changes were noted pre-versus post-therapy or between responders and non-responders (33). Consistently, a randomized controlled trial found that patients had a significant reduction of abdominal pain after PENFS therapy for 3 weeks, compared with patients with sham stimulation (20). A study consisting of 292 children aged 8–18 years with children’s gut-brain interaction disorder revealed that 3 weeks of PENFS therapy was associated with significant declines in abdominal pain index, nausea severity scale, and functional disability inventory score (34).

The Second Affiliated Hospital of Guangzhou University of Chinese Medicine conducted a clinical comparative study on patients with IBS-D. The results showed that after treatment with taVNA, the IBS-SSS, Hamilton Depression Scale (HAMD), and Hamilton Anxiety Scale (HAMA) of the patients significantly decreased (P < 0.05) (35).

The ear is the only part of the human body where the afferent vagus nerve is distributed on the surface. In the studies by Shi and Liu, it was clearly stated that the electrode stimulation site was located in the auricular region, namely the inner concave area of the ear lobe. By stimulating the vagus nerve in the auricular region, the central autonomic nervous system and the gastrointestinal system can be regulated, achieving cross-system effects such as improvement of gastrointestinal motility, inhibition of inflammation, and regulation of pain perception. This is in line with the traditional Chinese medicine ear acupuncture treatment in our country. In traditional Chinese medicine theory, “the stomach” and “the sympathetic nerve” and other acupoints are also concentrated in the inner and concave areas of the ear lobe (such as auricular cymba concha and cavity of concha). Traditional Chinese medicine believes that there is a specific association between ear points and the functions of the entire body’s internal organs (the theory of meridians and internal organs correspondence). By stimulating the ear points, the functions of the corresponding internal organs (such as the stomach and large intestine) can be regulated, alleviating symptoms related to IBS (27, 31, 36). In the review by Huang, we can see that by applying auricular acupressure to the corresponding auricular points of the large intestine, spleen, and liver, it is possible to regulate the functions of the internal organs, eliminate dampness, and stop diarrhea. IBS-SSS and the degree of anxiety have been significantly reduced in patients with IBS-D (37). It provides a new idea for the combination of traditional Chinese and Western medicine therapies.

At present, it is not clear what the absolute or relative contraindications of vagus nerve stimulation are. VNS showed a good safety profile in trials, with most patients reporting only minor side effects such as ear discomfort, mild skin irritation, or headache, which gradually disappeared after treatment ended (38).

However, there are indeed individual differences in the efficacy of vagus nerve stimulation therapy. Several possible reasons for this include: 1. Differences in stimulation parameters: variations in stimulation frequency (1Hz vs 25Hz), intensity, pulse width, treatment duration, and stimulation site (e.g., left auricular cavity vs both ears) can lead to differences in efficacy. For instance, 1Hz taVNS is more effective for migraines than 25Hz; taVNS and transcutaneous auricular nerve field stimulation (PENFS) differ in their targeting of the vagus nerve due to differences in electrode design and frequency parameters, which affect the efficacy (4, 23, 25). 2. Differences in individual anatomy and nerve innervation: There is controversy over the colon innervation range of the human vagus nerve, and there are individual differences in the distribution of the auricular vagus nerve and the baseline level of vagus nerve activity, resulting in different intensities of the effect of VNS on the gut and the activation of pathways; there is no morphological difference in the dimension of the vagus nerve, but functional activity differences may affect the efficacy (10, 15, 31). 3. Differences in disease subtypes and pathological states: The efficacy of VNS for IBS-C is clear, but there is still a lack of clear efficacy evidence for IBS-D and IBS-M. Disease subtypes are the core difference factors; in addition, individual differences in baseline inflammatory levels (TNF-α, IL-6 concentrations), visceral sensitivity thresholds, and intestinal barrier function (expression of tight junction proteins) will affect the anti-inflammatory and regulation of intestinal function effects of VNS (4, 18, 25). 4. Differences in the composition of the gut microbiota: Differences in the composition of the individual gut microbiota (such as the abundance of the Blautia genus) will affect the effect of VNS on regulating intestinal function through the microbiota-gut-brain axis. For example, after VNS treatment, the abundance of the Blautia genus in “excellent responders” of IBS is significantly higher than that in non-responders, and Blautia can maintain intestinal homeostasis by producing short-chain fatty acids and enhance the treatment response (10, 33). 5. Differences in the function of the central nervous system and the brain-gut axis: Differences in the degree of dysfunction of the brain-gut axis (such as the activity of the central pain processing pathway) and functional differences in pain-regulating brain regions will lead to different analgesic and emotional regulation effects of VNS (23, 39). 6. Differences in the drugs used simultaneously and basic health conditions: Some patients may receive VNS while concurrently using 5-aminosalicylic acid, biologics, etc., which may have synergistic or antagonistic effects with VNS; age, severity of comorbid mental disorders (anxiety, depression), and the degree of autonomic nerve dysfunction will also affect the regulatory effect of VNS on the brain-gut axis (3, 4, 23).

## 5 Study limitations

The existing clinical trials are mostly single-center and small-sample studies, which have problems such as short follow-up periods, insufficient data on long-term efficacy and recurrence rates, and are centered around IBS-C patients. The blind design of some studies is also not perfect, with significant differences between the stimulation sites of false stimulation and true stimulation, which may lead to patient blinding. The in-depth analysis of relevant signaling pathways (such as the downstream molecular mechanism mediated by α7nAChR) is insufficient. In terms of clinical application, non-invasive VNS lacks authoritative treatment approval; invasive iVNS is limited in clinical application due to surgical risks and costs.

## 6 Future research directions

Although VNS has shown great potential in the treatment of IBS, more long-term and systematic clinical trials are still needed to further validate its efficacy and safety. The core issues that need to be focused on in future research are optimal stimulation parameters for different IBS subtypes, combined regimens of VNS with other therapies (e.g., low FODMAP diet, prokinetics, and antidepressants), key targets to be validated in mechanistic studies of IBS-D, and so on.

## 7 Conclusion

In summary, by regulating GBA, reducing inflammatory response, and improving intestinal motility and visceral hypersensitivity, VNS significantly improved the symptoms of patients with IBS, indicated by decreased scores of VAS and IBS-SSS and increased CSBMs/weeks, BSFS, and IBS-QOL. In the future, the treatment of IBS will no longer rely on low-quality drugs and dietary therapies. Instead, it can mainly involve VNS, combined with drugs such as 5-aminosalicylic acid and biological agents for treatment. This will significantly improve the quality of life of patients with IBS.
