# Supplementary material for: Efficacy and mechanisms of vagus nerve stimulation in irritable bowel syndrome: a comprehensive literature review
Source: Front Immunol. 2026 Mar 11;17:1769070. doi: 10.3389/fimmu.2026.1769070 (PMC13013011; doi:10.3389/fimmu.2026.1769070)
Supplement: Supplementary file 1 [file DataSheet1.doc]

**Supplementfile 1**

**Search formula:**

((((((((((Vagus Nerve Stimulation) OR (Nerve Stimulations, Vagus)) OR (Nerve Stimulation, Vagus)) OR (Stimulations, Vagus Nerve)) OR (Stimulation, Vagus Nerve)) OR (Vagus Nerve Stimulations)) OR (Vagal Nerve Stimulation)) OR (Nerve Stimulations, Vagal)) OR (Nerve Stimulation, Vagal)) OR (Stimulations, Vagal Nerve)) OR (Stimulation, Vagal Nerve)) OR (Vagal Nerve Stimulations) OR (Neurostimulation)) AND ((((((((((Irritable Bowel Syndrome) OR (Irritable Bowel Syndromes)) OR (Syndrome, Irritable Bowel)) OR (Syndromes, Irritable Bowel)) OR (Colitis, Mucous)) OR (Colitides, Mucous)) OR (Mucous Colitides)) OR (Mucous Colitis)) OR (Colon, Irritable)) OR (Irritable Colon))
